# 567 Distance to Burn Center Is Not Associated with Long-term Psychologic Outcomes Following Burn Injury

**DOI:** 10.1093/jbcr/irae036.201

**Published:** 2024-04-17

**Authors:** Crystal Dye, William B Risinger, Spencer Thompson, David Keeven, Jason Smith, Matthew Bozeman

**Affiliations:** University of Louisville School of Medicine, Louisville, KY; University of Louisville Department of Surgery, Louisville, KY; University of Louisville School of Medicine, Louisville, KY; University of Louisville Department of Surgery, Louisville, KY; University of Louisville School of Medicine, Louisville, KY; University of Louisville Department of Surgery, Louisville, KY; University of Louisville School of Medicine, Louisville, KY; University of Louisville Department of Surgery, Louisville, KY; University of Louisville School of Medicine, Louisville, KY; University of Louisville Department of Surgery, Louisville, KY; University of Louisville School of Medicine, Louisville, KY; University of Louisville Department of Surgery, Louisville, KY

## Abstract

**Introduction:**

Burn injury predisposes patients to significant psychologic morbidity including anxiety and depression. Adding to the burden of injury, patients often require transfer to specialized burn centers located far from home, family, and friends. We sought to assess the impact of distance to burn center on long-term psychologic outcomes.

**Methods:**

Patients admitted to our American Burn Association verified center from January 2021- June 2023 were identified. Demographics, burn characteristics, and follow up anxiety (Generalized Anxiety Disorder-7) and depression (Patient Health Questionnarie-2) screening scores were reviewed. Distance to burn center (miles) was determined based on patient’s home address. Wilcoxson rank-sum, chi-squared, and logistic regression tests were used to identify variables associated with post-burn anxiety. Linear regression was used to evaluate the relationship between distance to burn center and GAD-7 scores.

**Results:**

Of the 267patients identified, 35.6% and 27.9% screened positive for anxiety (GAD-7 > 10) and depression (PHQ-2 > 3) respectively at follow-up. On univariate analysis, female sex (38% vs. 21%, p < 0.01), morphine milligram equivalents (MME) on the last day of hospitalization (30 vs. 22.5, p=0.02), prior psychiatric history (37% vs. 13%, p< 0.01), and inhalation injury (9% vs. 3%, p=0.05) were associated with positive anxiety screens. Distance to burn center was similar between patients with positive and negative anxiety screens (40 vs. 47 miles, p=0.49). Via logistic regression, MME on the last day of hospitalization (OR: 1.02, p=0.03) and a history of psychiatric illness (OR: 3.93, p < 0.01) all predicted post-injury anxiety when accounting for age, %TBSA, burn mechanism, sex, injury at workplace, inhalation injury, and hand/face involvement. Distance to burn center showed no linear relationship to GAD-7 scores (β = -0.004, R2=0.00, p=0.64).

**Conclusions:**

Distance to burn center is not associated with increased long-term psychologic outcomes following burn injury, further supporting the transfer of patients to specialized burn centers. However, prior history of psychiatric illness and increased utilization of inpatient narcotics are all associated with increased probability of long-term anxiety.

**Applicability of Research to Practice:**

Provides further evidence for the transfer of burn patients to specialized centers.

